# Influence of temperature change in donor corneas on postoperative endothelium cell density following endothelial transplantation

**DOI:** 10.1038/s41598-020-57614-6

**Published:** 2020-01-20

**Authors:** Koji Kakisu, Takefumi Yamaguchi, Jun Shimazaki

**Affiliations:** Department of Ophthalmology, Tokyo Dental College Ichikawa General Hospital, Chiba, Japan

**Keywords:** Corneal diseases, Outcomes research

## Abstract

To examine the influence on postoperative endothelial cell density (ECD) caused by preoperative temperature change from 4 °C to room temperature in donor corneas for Descemet stripping automated endothelial keratoplasty (DSAEK). This retrospective, case-controlled comparison of 100 eyes that underwent DSAEK using imported donor corneas transferred from an overseas eye bank (SightLife, Seattle, WA, USA). Fifty donor corneas experienced temperature reversal for ECD measurement (TR group), and postoperative outcomes were compared with 50 disease-matched cases that did not experience temperature changes before DSAEK (NTR group). The main outcome measure was endothelial cell loss and reduction rate of ECD at 1, 3, and 6 months following DSAEK. ECD at 3 months following DSAEK was significantly less in the TR group (1458 ± 494/mm^2^) than in the NTR group (1696 ± 374/mm^2^; *P* = 0.014), though ECD at 6 months was not significantly less in the TR group. The reduction rate of ECD at 3 months was greater in the TR group (42.3% ± 17.2%) than in the NTR group (35.7% ± 14.2%; *P* = 0.044), though reduction rate of ECD at 6 months was not significantly less in the TR group. We found that preoperative temperature change in donor corneas may adversely affect ECD following DSAEK.

## Introduction

Recently, Descemet stripping automated endothelial keratoplasty (DSAEK) has become the leading procedure for the treatment of bullous keratopathy; however, postoperative decreases in endothelial cell density (ECD) remain a major concern. A number of potential causes for ECD loss have been reported, including longer death-to-preservation time^1,2^, extended preservation time^1^, preoperative iris damage in the recipient^3^, intraoperative complications^4^, and air injection into the anterior chamber^5^. To assess the tissue for endothelial damage and to examine the endothelial cell density, imported donor corneas shipped to Japan were rewarmed to room temperature from 4 °C preservation for ECD measurements, then preserved at 4 °C prior to surgery. We have experienced cases with relatively high ECD loss after DSAEK when using imported postprecut donor corneas. While seeking the reason for ECD loss, we speculated that preoperative temperature change may be responsible in corneas that required temperature reversal during tissue re-evaluation prior to surgery. Because of this concern, donor endothelial examination was discontinued as of January 2015. To study the effect of preoperative temperature changes on postoperative ECD, we conducted this case-control study.

## Methods

We conducted a single-center, retrospective, case-control study, involving 100 eyes that underwent DSAEK using imported postprecut donor corneas transferred from an overseas eye bank (SightLife, Seattle, WA, USA) in Tokyo Dental College between June 2010 and December 2016. No organs were procured from prisoners. The use of imported donor corneas was approved by the Ethical Committee of Tokyo Dental College and was performed in accordance with the tenets set forth in the Declaration of Helsinki (I 18–19). Our institutional review board waived the requirement for informed consent for this retrospective study. Patient data was anonymized before access and analysis. The original diseases for DSAEK included pseudophakic bullous keratopathy (PBK), post-laser iridotomy bullous keratopathy, Fuchs endothelial corneal dystrophy (FECD), endothelium decompensation following corneal transplantation, or pseudoexfoliation syndrome (Table 1). Patients with a minimum of 6 months of follow-up were included. Donor corneas were requested 2 weeks before surgery and were typically transported to our hospital at 7:00 p.m. to 8:00 p.m. on the day before surgery. The corneas were rewarmed to room temperature to examine possible changes in the corneal endothelium for half a day by eye bank in Tokyo Dental College Ichikawa General Hospital; corneas were then preserved at 4 °C prior to surgery. We studied postoperative outcomes in 50 consecutive DSAEK procedures using donor corneas that underwent temperature reversal from February 2015 to December 2016 (TR group). The results were compared with 50 disease-matched cases that did not undergo temperature changes from October 2010 to January 2015 (NTR group). Clinical data were obtained from medical records. The primary outcome measure of this study was ECD at 1, 3, and 6 months postoperatively. The following factors were also analyzed and compared between the two groups:

1. Donor-related factors, including age, sex, preoperative ECD, death-to-preservation time, death-to-operation time, central graft thickness, and graft size.
2. Recipient-related factors, including age, sex, central corneal thickness, original diseases, and best spectacle-corrected visual acuity (BSCVA).

**Table 1:** Demographic profiles in temperature reversal (TR) and non-temperature reversal (NTR) groups.

	T-R (+) (n = 50)	T-R (−) (n = 50)	P value
**Characteristics**			
Mean Age, year (±SD)	74.4 (8.2)	75.5 (6.9)	0.46
Gender (%) Male Female	17 (34.0) 33 (66.0)	12 (24.0) 38 (76.0)	0.28
Central corneal thickness (*μ*m) (±SD)	746.6 (105.1)	731.9 (120.2)	0.52
**Indication, n (%)**			
Pseudophakic BK	15 (30)	15 (30)	
After laser iridotomy	14 (28)	14 (28)	
FECD	12 (24)	12 (24)	
Endothelium decompensation after transplantation	8 (16)	8 (16)	
Pseudoexfoliation syndrome	1 (2)	1 (2)	
**ECD at 3months Surgeons**			
A	1513 (23)	1757 (20)	0.10
B	1713 (9)	1656 (15)	0.74
C	1237 (8)	1629 (7)	0.23
D	1187 (3)	1673 (6)	0.10
E	1343 (3)	1834 (1)	
F	1392 (4)	1560 (1)	
**Graft size** (**mm)**			
7	1209 (1)	None	
7.75	1369 (4)	1576 (3)	0.72
8.0	1436 (29)	1696 (27)	0.051
8.25	1887 (8)	1828 (8)	0.82
8.5	1573 (7)	1671 (7)	0.57
8.75	none	1895 (2)	

SD: standard deviation; BK: bullous keratopathy; FECD: Fuchs endothelial corneal dystrophy.

### Measurement of corneal endothelial cell density

The count of ECD after shipping was evaluated using donor corneas in the vial by the center method with specular microscopy (EKA-10; Konan Medical, Nishinomiya, Japan). A minimum of 100 cells were counted at the central area. Postoperative measurements of ECD were performed using a noncontact specular microscope (EM-4000, TOMEY; Nagoya, Japan) at 1, 3, and 6 months postoperatively.

### Visual acuity

Visual acuity was measured using the Snellen visual acuity chart; we analyzed the results using logarithm of the minimum angle of resolution (logMAR) equivalent units, including manifest refraction, spherical equivalent, and cylindrical error.

### Surgical technique

Six experienced surgeons were involved in the DSAEK procedures. Descemet’s membrane and diseased endothelium were stripped from the planned graft area using the reverse Sinskey hook (ASICO, Westmont, IL, USA). Venting incisions were made to improve graft adherence. A 4.5–5-mm clear corneal incision was created using a slit knife. The donor cornea was cut with a diameter of 7.0–8.75 mm, depending on the recipient corneal diameter. The trephinated donor lenticule was inserted into the recipient anterior chamber using a Busin glide (ASICO, Westmont, IL, USA). Once the donor graft was properly unfolded and well centered, air was injected into the anterior chamber. After the anterior chamber was filled completely with air for 10–15 min, the air was reduced to prevent pupillary block. No peripheral iridectomy was performed. Following DSAEK, each patient received a topical application of antibiotics (1.5% levofloxacin, Santen Pharmaceutical Co., Osaka, Japan) and 0.1% dexamethasone (Sanbetasone, Santen Pharmaceutical Co.) eye drops, five times daily; the number of daily administrations was then tapered over the following several months.

### Statistical analysis

The ECD and reduction rate of ECD were expressed as either mean ± standard deviation (SD) or range, and were compared using an unpaired Student’s t-test. Comparisons between categorical variables were analyzed via the chi-squared test. *P*-values less than 0.05 were considered statistically significant.

## Results

### Recipient background

The demographics of all patients are summarized in Table 1. There were no significant differences in baseline characteristics between the TR and NTR groups, such as recipient age (*P* = 0.46), sex (*P* = 0.28), or central corneal thickness (*P* = 0.52).

### Donor background and graft condition

In the TR and NTR groups, the respective mean donor ages were 62.4 ± 9.2 and 60.4 ± 10.3 years; the respective mean ECD values were 2604 ± 316 cells/mm^2^ and 2652 ± 278 cells/mm^2^; and the respective mean death-to-operation times were 6.1 ± 1.1 and 6.9 ± 0.8 days (Table 2). There were no significant differences between the two groups.

**Table 2:** Donor-related conditions and surgical procedures in temperature reversal (TR) and non-temperature reversal (NTR) groups.

	TR group (n = 50)	NTR group (n = 50)	P value
Mean age, year (±SD)	62.4 (9.2)	60.4 (10.3)	0.30
**Sex (%)**			
Male	35	33	0.53
Female	15	17	
ECD (cells/mm^2^)	2604 ± 316	2652 ± 278	0.41
Death to preservation (minutes)	681 ± 323	701 ± 299	0.74
Death to operation (days)	6.9 ± 1.1	6.9 ± 0.8	0.83
Graft thickness (µm)	143 ± 30	139 ± 19	0.55
**Graft size (mm)**			
7	1	0	
7.75	3	1	
8	19	22	
8.25	7	4	
8.5	7	7	
8.75	0	1	1.0
Unknown	0	2	
Solitary DSAEK	40 (80%)	40 (80%)	
DSAEK + PEA + IOL	6 (12%)	9 (18%)	0.40
DSAEK + IOL suturing	4 (8%)	1 (2%)	0.17

ECD: endothelial cell density; PEA: phacoemulsification and aspiration; IOL: intraocular lens; DSAEK: Descemet stripping automated endothelial keratoplasty.

### Graft survival rate at 6 months

Graft survival rates at 6 months were 94.0% (47 eyes) and 100% (50 eyes) in the TR and NTR groups, respectively (*P* = 0.08). Reasons for graft failure in the TR group included endothelial rejection (n = 1), endothelium decompensation (n = 1), and herpetic keratitis (n = 1).

### Visual acuity

Although the mean preoperative BCVA in the NTR group tended to be better than in the TR group, there were no significant differences between the two groups (1.26 ± 0.49 vs. 1.07 ± 0.60 logMAR, respectively; *P* = 0.084). In the TR group, 42 eyes (91.3%) exhibited improved vision at 6 months postoperatively. One eye experienced a decline in vision due to the recurrence of herpetic keratitis; an eye with FECD showed slow visual recovery due to an unknown cause. In the NTR group, 38 eyes (92.7%) exhibited improved vision at 6 months postoperatively. Two eyes (5.3%) experienced a decline in vision due to subepithelial opacification or slow visual recovery from an unknown cause. Postoperative BSCVA did not significantly differ between the two groups (0.69 ± 0.49 vs. 0.55 ± 0.59 at 1 month, *P* = 0.14; 0.38 ± 0.37 vs. 0.44 ± 0.40 at 3 months, *P* = 0.46; 0.32 ± 0.35 vs. 0.36 ± 0.32 at 6 months, *P* = 0.59).

### Changes in ECD

There were no significant differences in either surgeons or graft diameters between the TR + and TR- groups. The mean preoperative ECD was similar between the two groups (*P* = 0.41) (Table 3). Postoperative ECD was higher in the NTR group up to 6 months; the ECD at 3 months showed a significant difference (*P* = 0.014), though ECD at 6 months was not significantly less in the TR group. The reduction rate of ECD at 3 months revealed a significant difference (*P* = 0.044), though reduction rate of ECD at 6 months was not significantly less in the TR group. The mean preoperative ECD was similar between the two groups without a history of trabeculectomy or postoperative re-bubbling to exclude the factor of preoperative iris damage or postoperative toxicity of air (*P* = 0.93). The ECD at 3 months showed a significant difference (*P* = 0.017). The reduction rate of ECD at 3 months revealed a significant difference (*P* = 0.012) (Table 4).

**Table 3:** Postoperative endothelial cell (EC) counts and reduction of endothelial cell density (ECD) in temperature reversal (TR) and non-temperature reversal (NTR) groups.

Time	ECD (cells/mm^2^) Mean (±SD)				P value	ECD loss (%) Mean (±SD)		P value
	TR group	N	NTR group	N		TR group	NTR group	
Baseline	2604 (316)	50	2653 (278)	50	0.41			
1 month	1619 (554)	35	1740 (463)	43	0.30	38.2 (20.8)	33.7 (19.5)	0.33
3 months	1458 (494)	38	1696 (374)	46	0.014	42.3 (17.2)	35.7 (14.2)	0.044
6 months	1347 (448)	36	1525 (587)	36	0.15	48.7 (15.7)	42.2 (22.1)	0.15

**Table 4:** Postoperative endothelial cell counts and reduction of endothelial cell density (ECD) in temperature reversal (TR) and non-temperature reversal (NTR) groups without trabeculectomy or re-bubbling.

Time	ECD (cells/mm^2^) Mean (±SD)				P value	ECD loss (%) Mean (±SD)		P value
	TR group	N	NTR group	N		TR group	NTR group	
Baseline	2630 (308)	41	2635 (288)	41	0.93			
1 month	1646 (521)	27	1803 (395)	35	0.23	37.4 (21.7)	30.0 (19.5)	0.16
3 months	1446 (521)	31	1716 (395)	38	0.017	44.5 (18.6)	34.3 (14.2)	0.012
6 months	1378 (437)	29	1584 (580)	31	0.13	47.7 (15.6)	39.7 (22.8)	0.11

## Discussion

There is an existing concern regarding postoperative ECD loss following endothelial keratoplasty. Recently, Dickman *et al*. reported that rates of ECD loss were comparable between eyes that underwent penetrating or endothelial keratoplasty (EK)^6^. Particularly, ECD reductions during early postoperative periods are relatively high, regardless of improvements in surgical techniques. There are numerous factors that may potentially affect ECD loss during early postoperative periods following EK. Kitazawa and associates recently reported that donor corneas contained a significant number of dead endothelial cells, and that the presence of these cells may be responsible for early declines in ECD following DSAEK^7^. Their report indicated that proper management of donor corneas is key to successful long-term preservation of the corneal endothelium following DSAEK.

As a leading hospital for corneal transplantation in Japan, we have performed many DSAEK procedures using imported donor corneas. We were particularly concerned about the long distance transportation and following lamellar dissection procedures. We and others reported that the average rate of ECD loss ranged from 1.75% to 3.7% after precut procedure, and 2.3% to 3.79% for overseas transportation^7,8^. Although these changes were statistically significant, the impacts on clinical outcomes seemed to be relatively low. Through discussion with eye bank members and other researchers, we realized that additional manipulation of donor corneas, particularly temperature changes, might adversely affect donor corneal endothelium. In the present case-control study, we observed that the use of donor corneas that experienced temperature reversal resulted in statistically significant decreases in ECD following DSAEK. The mean ECD loss at 3 months postoperatively was 44.5% for the TR group and 34.3% for the NTR group. The difference of the mean endothelial cell loss in corneas with temperature changes was approximately 10%; this impact seemed greater than those caused by post lamellar dissection procedure or overseas transportation. There were no significant differences between the two groups in other conditions such as patient background, donor background, graft condition, or surgical procedures. Further, there were no differences in ECD between eyes with and without either a history of trabeculectomy or postoperative re-bubbling. It seems likely that temperature changes in donor corneas affected endothelial survival following DSAEK. Although we did not find significant differences in graft survival rate or BSCVA, the differences in ECD seemed to be at 3 months and at 6 months after DSAEK. It is assumed that the factors except for donor corneas had a great influence on ECD at 6 months. Long-term observation of these cases may find worse graft survival in eyes that underwent DSAEK using donor corneas with TR. Although the exact mechanism underlying the association between temperature changes and ECD loss remains unclear, we speculate that the reactive oxidative stress (ROS) may play a role. Recent studies have reported that ROS induces apoptosis in corneal endothelial cells^9^. Rauen^10^ *et al*. reported that during exposure to 4 °C, and to a lesser extent during rewarming, cultured corneal endothelial cells incurred injury mediated by ROS^9^. This injury was accompanied by pronounced morphologic alterations that occurred predominantly during rewarming, representing apoptotic features.

In conclusion, the limitations of this study include the small patient population, and the short duration of follow-up time. As the reason for small population, ethically unacceptable to increase the number of cases for the possibility of affecting ECD, retrospective study though further clinical and basic investigations are needed to elucidate the long-term impact on graft survival and improvement in endothelial preservation. The findings of this present study showed that preoperative temperature change in donor corneas may be related to increased ECD loss after DSAEK.
